# Heroin and its metabolites: relevance to heroin use disorder

**DOI:** 10.1038/s41398-023-02406-5

**Published:** 2023-04-08

**Authors:** Michele Stanislaw Milella, Ginevra D’Ottavio, Silvana De Pirro, Massimo Barra, Daniele Caprioli, Aldo Badiani

**Affiliations:** 1 Toxicology Unit, Policlinico Umberto I University Hospital, Rome, Italy; 2 Laboratory affiliated to the Institute Pasteur Italia-Fondazione Cenci Bolognetti–Department of Physiology and Pharmacology, Sapienza University of Rome, Rome, Italy; 3 Santa Lucia Foundation (IRCCS Fondazione Santa Lucia), Rome, Italy; 4 Norwegian Centre for Addiction Research (SERAF), Faculty of Medicine, University of Oslo, Oslo, Norway; 5 Sussex Addiction and Intervention Centre (SARIC), School of Psychology, University of Sussex, Brighton, UK; 6 Fondazione Villa Maraini, Rome, Italy

**Keywords:** Addiction, Pharmacodynamics

## Abstract

Heroin is an opioid agonist commonly abused for its rewarding effects. Since its synthesis at the end of the nineteenth century, its popularity as a recreational drug has ebbed and flowed. In the last three decades, heroin use has increased again, and yet the pharmacology of heroin is still poorly understood. After entering the body, heroin is rapidly deacetylated to 6-monoacetylmorphine (6-MAM), which is then deacetylated to morphine. Thus, drug addiction literature has long settled on the notion that heroin is little more than a pro-drug. In contrast to these former views, we will argue for a more complex interplay among heroin and its active metabolites: 6-MAM, morphine, and morphine-6-glucuronide (M6G). In particular, we propose that the complex temporal pattern of heroin effects results from the sequential, only partially overlapping, actions not only of 6-MAM, morphine, and M6G, but also of heroin per se, which, therefore, should not be seen as a mere brain-delivery system for its active metabolites. We will first review the literature concerning the pharmacokinetics and pharmacodynamics of heroin and its metabolites, then examine their neural and behavioral effects, and finally discuss the possible implications of these data for a better understanding of opioid reward and heroin addiction. By so doing we hope to highlight research topics to be investigated by future clinical and pre-clinical studies.

## Introduction

Heroin (3,6-diacetylmorphine or diamorphine) is a semi-synthetic derivative of morphine, a naturally occurring opiate contained, along with codeine, in the latex of the opium poppy (*Papaver somniferum*). The opium poppy was first domesticated circa 6000 B.C.E. in Europe, and its cultivation spread eastwards over the following millennia [1, 2]. It is worth noticing that the frequent reference in textbooks and journal articles to a supposed initial spread of opium production from Mesopotamia has long been shown to be based on flawed scholarship [2, 3]. While the medical use of opium in antiquity (first by Greco-Roman medicine and centuries later by Arab, Indian, and Chinese medicines) is well documented, reliable evidence of widespread ‘recreational’ use is much more recent and appears to be related to technological innovations. The introduction of tobacco and tobacco pipe by European traders in South-East Asia (16th-17th century) made it possible to smoke opium and opium laced tobacco [4], whereas the standardization of laudanum, a hydroalcoholic extract (tincture) of opium, in Europe (17th–18th century) made it possible to take large amounts of morphine and codeine with a single draught [5]. Technological innovations were crucial also in the case of morphine, which was isolated in 1817, but became widely used for medical and non-medical purposes only after the invention of the hypodermic syringe a few decades later [5]. At present, the non-medical use of morphine is a relatively rare occurrence, compared to heroin. The pharmacokinetics and pharmacodynamics of morphine, which will be discussed in the following sections, might help to explain the reason for this preference.

First synthesized in 1874 and then commercialized as a cough suppressant and controversial respiratory disease remedy [6], heroin readily emerged as a public health concern when it was found that, in addition to having a powerful analgesic effect, it could trigger severe dependence and illicit use [7]. In our days, despite decades of public awareness and the recent introduction of a whole new plethora of synthetic drugs, the attraction of heroin has not waned. Global, world-wide prevalence of opioid use disorder has progressively increased in the past 30 years (>40% in 2019 relative to 1990), albeit with stark regional differences, as indicated by dramatic surges in some countries (e.g., +351% in the USA, +308 in Sweden, +261% in Canada, +200% in Scotland) but not in others (e.g., −23% in Switzerland, −32% in Italy) [8]. Heroin is still the main opioid of abuse in most countries (with noticeable exceptions, such as the USA and Canada) [8, 9]. This is consistent with the parallel increase (>50%) in the global supply of heroin over the same time period, a figure that can be estimated with a certain degree of precision on the basis of opium production (monitored by satellite imagery) minus seizures [10]. Thus, it appears that the heroin issue is possibly even more relevant in the 21st century than it was in the previous one.

Given this premise, it is surprising that the psychopharmacology of heroin is still poorly understood, compared to that of other drugs of abuse, such as cocaine and psychostimulants in general. Since heroin is rapidly deacetylated to 6-monoacetylmorphine (6-MAM) and then to morphine, drug addiction literature has long settled on the notion that heroin is little more than a means to deliver morphine and/or 6-MAM to the brain [11, 12]. In contrast to these former views, we will argue here for a more complex interplay among heroin and its active metabolites, 6-MAM, morphine, and morphine-6-glucuronide (M6G), as it is unlikely that the multistage course of heroin effects could be produced by just one of these molecules. Although there is a dearth of data concerning the time course of the subjective effects of heroin, it is in fact commonly acknowledged by users (personal observations in people with heroin use disorder) and experts in the field [13–15] that intravenous (i.v.) heroin produces an almost instantaneous surge of ecstatic pleasure (often referred to as ‘flash’ or ‘rush’), lasting about one minute, followed by a pleasant state of stunned calm and detachment, and finally by a sense of well-being and contentment that persists for several hours. We propose that this pattern results from the sequential, only partially overlapping, actions not only of 6-MAM, morphine, and M6G, but also of heroin per se, which, therefore, should not be seen as a mere brain-delivery system for its active metabolites.

We will first review the literature concerning the pharmacokinetics and pharmacodynamics of heroin and its metabolites, and then examine their neural and behavioral effects. Finally, we will discuss the possible implications of these data for a better understanding of opioid reward and heroin addiction. By so doing we hope to highlight research topics to be investigated by future clinical and pre-clinical studies.

## Pharmacokinetics of heroin and its metabolites

**Fig. 1:**
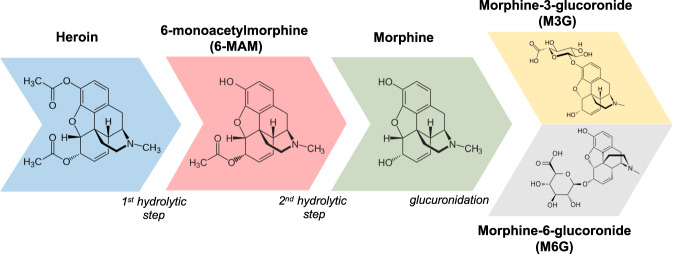
Molecular structure of heroin and its metabolites. Schematic representation of the metabolic pathway of heroin with the sequential breakdown into the main metabolites. The involved enzimatic processes are listed in italics.

The biotransformation of heroin and of its metabolites (Fig. 1) involves: (i) hydrolytic reactions, catalyzed by serum- or butyryl-cholinesterase in the plasma, and by carboxylesterases in the liver, brain, and other tissues; (ii) synthetic reactions, mainly glucuronidation in the liver (but also in the brain, kidney, and intestine), and to a lesser extent sulfation; (iii) oxidative reactions, yielding minor metabolites. The transfer rates of heroin and its metabolites across the blood-brain barrier mostly depends on their lipophilicity, which decreases at each metabolic step: heroin>6-MAM»morphine»M6G [16] (see Table 1). The transfer rates of morphine and M6G (but not that of heroin and 6-MAM) also depend on active transport by the P-glycoprotein and other efflux transporters [17, 18]. Quite obviously, quantitative data concerning the distribution of heroin and its metabolites to the human brain are very limited (heroin cannot be detected even post-mortem in the brain of people who died of heroin overdose [19]).

**Table 1:** Overview of the principal pharmacokinetic parameters and characteristics of heroin and its metabolites following an intravenous administration of heroin (120–450 mg) in humans.

	Heroin	6-MAM	Morphine	M6G	references
T_max_ (minutes)	0.3–1.5	0.3–2.7	3.6–7.8	42.6–119	[22, 25, 46, 47]
C_max_ (ng/ml)	1530–3960	1731–5742	340–829	700–1270	[25, 46, 47]
Clearance (L/h)	high 696–822	high 564–607			[22, 25, 46, 47]
Fraction excreted in urine (% of dose)	<1	1.5	10	55	[26, 264]
V_d_ (L)	37–96	325			[22, 25, 46, 47]
t_1/2_ (minutes)	very short 1.3–3.8	short 3–52	long 176–287	very long 267.5–681	[22, 25, 46, 47]
Partition coefficient	0.85	0.61	–0.07	–0,79	[16]

*6-MAM* 6-monoacetylmorphine, *M6G* morphine-6-glucuronide, *V*_*d*_ volume of distribution.

**Fig. 2:**
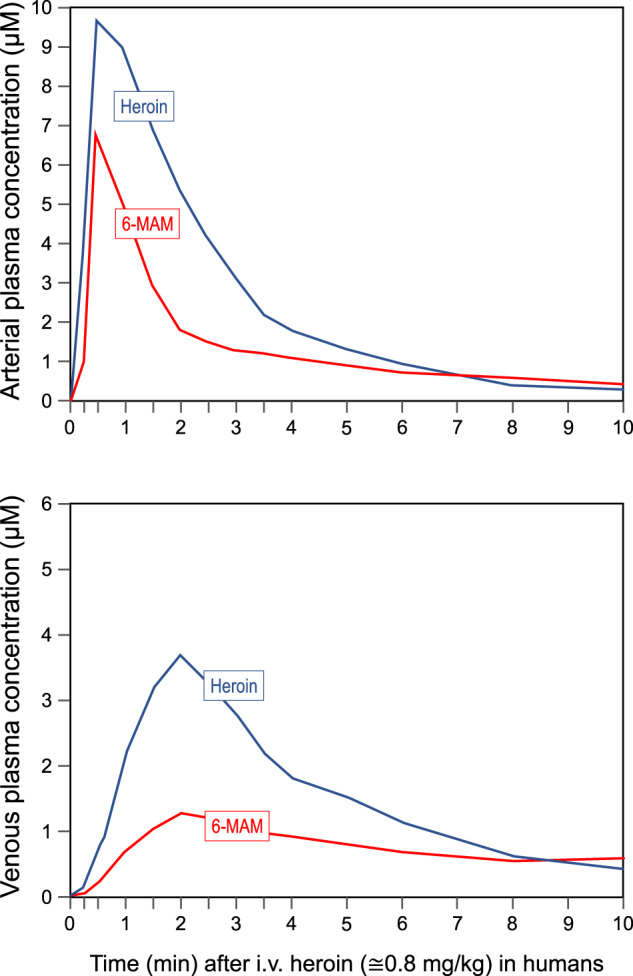
Early pharmacokinetic profile of heroin by i.v. administration and metabolite 6-MAM. Time course of arterial and venous concentrations of heroin (blue line) and 6-MAM (red line), after an i.v. injection of heroin (≅0.8 mg/kg) in humans. Based on data extrapolated from Rentsch et al. 2001 [22].

**Fig. 3:**
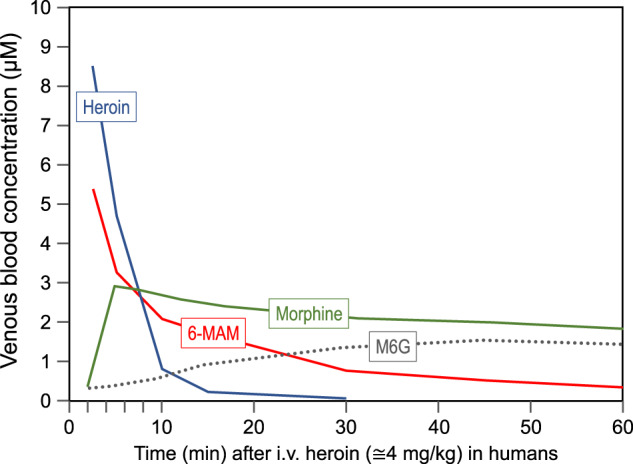
Pharmacokinetic profile of heroin by i.v. administration and its main metabolites. Time course of venous concentrations of heroin (blue line), 6-MAM (red line), morphine (green line), and M6G (dotted grey line), after an i.v. injection of heroin (≅4 mg/kg) in humans. Based on data extrapolated from Rook et al. 2006 [25].

**Fig. 4:**
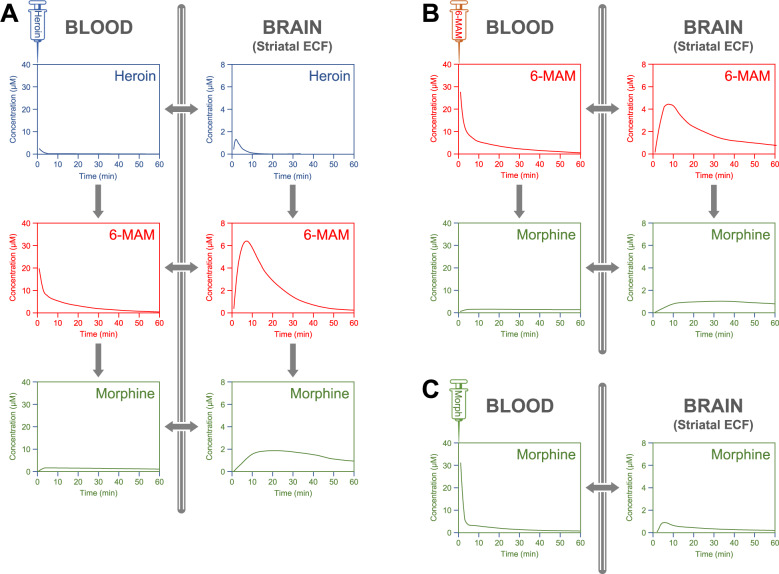
Concentration-time profiles of heroin and its metabolites in the rat blood and brain. Concentrations of heroin (blue), 6-MAM (red), and morphine (green) in the blood and in the striatal extracellular fluid of rats, after an i.v. injection of 1.3 µmol ($\cong$4 mg/kg) of heroin (**A**), 6-MAM (**B**), or morphine (**C**). Based on data extrapolated from Gottås et al. 2014 [20] and supplementary data (same animals of Fig. 3). Notice that in this study M6G was not quantified, as in the rat, under normal conditions, the synthesis of this metabolite is negligible.

More information about the brain distribution of heroin and its metabolites is available for rodents, as their brain concentrations were quantified after i.v. administration of heroin in the rat [20] and transfer rate constants were estimated after subcutaneous administration in the mouse [21]. However, caution should be applied in extending data collected in rodents to humans, given the much faster metabolism of heroin in mice and rats relative to humans (compare Figs. 2–4).

### Pharmacokinetics of heroin

The pharmacokinetics of heroin will be discussed for each route of administration.

#### Intravenous injection

Following i.v. injection in humans, heroin plasma concentrations peak (C_max_) almost immediately (T_max_ ≅ 30 s in the arterial circulation; T_max_ ≅ 2 min in the venous circulation) [22] (Fig. 2), and then decline steeply with a half-life (t_1/2_) of 3–4 min [22–25]. Within 10–45 min, heroin becomes undetectable in the blood [24–27] (Fig. 3). The blood clearance rate for heroin (128–1920 L/h) exceeds the rate of hepatic blood flow [26], and renal clearance accounts for less than 1% of the administered dose [27]. Indeed, heroin is rapidly converted to 6-MAM by plasma cholinesterases and by the carboxylesterases present in the liver, kidney and other organs [28, 29]. Spontaneous, non-enzymatic hydrolysis may also occur [30]. However, it is important to emphasize that for the first 8 min after i.v. injection heroin concentrations in both arterial and venous circulation remain higher than that of all other active metabolites, including 6-MAM (Figs. 2 and 3). Thus, there is no reason to discard a major role of heroin per se in the early response to heroin in humans, contrary to the widely held notion of heroin as a mere pro-drug.

In the rat, after i.v. administration, heroin peaks very rapidly in the brain (T_max_ = 1.5–2 min in the striatal extracellular fluid), where is then metabolized by esterases (t_1/2_ = 1–2 min) [20, 31], and becomes undetectable within 10–30 min (Fig. 4).

#### Inhalation

Heroin can be inhaled by ‘chasing the dragon’ (where the users heat the drug over aluminium foil and inhale the resulting fumes) or by smoking tobacco laced with heroin. The ‘rush’ provided by this route of administration is comparable to that of the i.v. route [32], but its popularity varies greatly, mainly as a function of the prevailing form of street heroin available in any given region. Heroin hydrochloride (the prevailing form of street heroin in most regions of the USA) is not suitable to this route of administration because most of it is destroyed at the temperatures required for vaporization. In contrast, freebase heroin (like the brown heroin popular in Europe) vaporizes at relatively gentle heat [33].

There is relatively scarce information about the pharmacokinetics of heroin after inhalation. Absorption is extremely rapid owing to the lipophilic structure of heroin, even though its alkaline pK_a_ (7.95) results in the predominance of the ionized form in the acidic alveolar subphase fluid (pH ≈ 6.9; [34]). Bioavailability has been estimated to be 38–53% when heroin is taken by ‘chasing the dragon’ [24, 25, 35] and about 14% when heroin is taken by smoking laced tobacco [35]. Unsurprisingly, the C_max_ is considerably lower than after i.v. injection [25] with a T_max_ up to 5 min [24]. The t_1/2_ is about the same for the two techniques of inhalation (3–4 min) [24, 25].

#### Insufflation

The rich submucosal venous plexus of the nose and the fenestrated endothelia of its capillaries allow for the fast absorption of heroin after insufflation, although, to the best of our knowledge, there are no data on its bioavailability [36]. However, only a fraction of the dose is actually absorbed, as heroin can be hydrolysed in the nasal cavity by a variety of enzymes [37, 38]. Also in this case the C_max_ is much lower and the T_max_ longer (about 4–5 min) than after i.v. injection [39–41]. This explains why ‘snorters’ do not achieve the same level of euphoria experienced by ‘mainliners’, or even smokers [39]. The t_1/2_ is slightly higher of that seen after i.v. injection (5–6 min) [40, 41]. A particular type of intranasal delivery device is represented by the nasal spray, developed as an alternative to injectable diacetylmorphine for replacement treatments (see Conclusions) [42].

#### Intramuscular and subcutaneous injection

The subcutaneous and intramuscular injection of heroin is often due to poor injection practice or to the inability to find a patent vein [43, 44]. However, heroin users who wish to lengthen the duration of drug effects and to experience a calm, warm ‘high’ rather than the ‘rush’ might deliberately inject the drug intramuscularly [43, 45]. Heroin metabolism in the muscle is in fact negligible, and the C_max_ is double of that seen after insufflation [41]. Furthermore, heroin is slowly released from the muscle into the general circulation, resulting in a half-life considerably longer (t_1/2_ = 7.8 min [46]) than after i.v. injection.

#### Oral administration

After oral administration, no heroin or 6-MAM can be detected in the blood (because of hydrolysis in the duodenum and colon, and virtually complete hepatic first-pass effect), whereas circulating morphine reaches significant levels (about 80% of that measured after the administration of an equal dose of morphine, but with shorter T_max_) [23, 47]. This route of administration has been recently employed for the administration of medical grade heroin as a replacement treatment in people with heroin use disorder [48] (see Conclusions).

### Pharmacokinetics of 6-MAM

After intravenous administration of heroin, 6-MAM peaks at more or less the same time of heroin both in the venous and in the arterial circulation (Fig. 2). The C_max_ is similar to that of heroin in the arterial circulation but considerably lower in the venous circulation [22, 25, 46] (see Figs. 2 and 3). As detailed in the previous section, plasma concentrations of 6-MAM remain lower than that of heroin for the first 8 min after i.v. injection. The t_1/2_ of 6-MAM is longer than that of heroin, although estimates vary greatly from study to study (3–52 min), and can be detected in the plasma for hours, at a time when heroin has already disappeared [24, 25, 46, 47]. With other routes of administration the T_max_ of 6-MAM is considerably longer [39–42].

In contrast, i.v. injection of heroin in the rat results in peak plasma and striatal concentrations of 6-MAM much higher than those of heroin, with a T_max_ of 2 min in the venous blood and 8 min in the striatum [20] (Fig. 4). This is likely due to inter-species differences in esterase activity [49]. Given its high lipophilicity, 6-MAM passively diffuses across the blood-brain barrier [50]. It has been proposed, based on data from a subcutaneous injection of heroin in mice, that the rapid increase in 6-MAM brain concentration is mainly due to the deacetylation of heroin in the blood, before its entry into the brain [21]. Accordingly, vaccine-generated antibodies targeting heroin and its metabolites reduce 6-MAM concentration in the brain, without affecting that of heroin [51]. However, the striatal C_max_ of 6-MAM after heroin administration in the rat is about 50% higher than after equimolar doses of 6-MAM [31], indicating that, at least in the rat, a significant fraction of brain 6-MAM results from the local deacetylation of heroin.

The second hydrolytic step in the metabolism of heroin mostly depends on liver carboxylesterase-2, which deacetylates 6-MAM to morphine [52].

### Pharmacokinetics of morphine

Following heroin i.v. administration in humans, morphine plasma levels rise quickly (Fig. 3) with a T_max_ ranging between 4 and 8 min [25, 47]. The T_max_ after intranasal or intramuscular injection of heroin is considerably longer, ranging between 10 and 90 min [40, 41, 46]. Plasma levels decline at a much slower pace than for heroin or 6-MAM, with a t_1/2_ of about 3–4 h [25, 47] (Table 1). The T_max_ and t_1/2_ after morphine administration have similar values [53, 54]. Systemic clearance is high (75–118 L/h) [26], which indicates a high hepatic extraction ratio [55]. Morphine is rapidly distributed to highly perfused organs and tissues. However, its distribution to the brain is limited by the hydrophilic structure and by a pK_a_ = 8.2, resulting in the prevalence of the ionized form at physiological pH [26]. Moreover, morphine is a substrate for the efflux transporter P-glycoprotein and other probenecid-sensitive transporters [56, 17]. These mechanisms greatly reduce the ability of morphine to distribute to the brain.

**Fig. 5:**
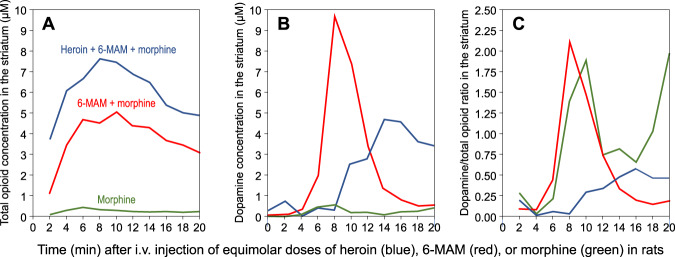
Comparative analysis of total opioid and dopamine concentrations in the rat striatum. Striatal concentration of opioids and dopamine in rats that had received an i.v. injection of 1.3 µmol (≅4 mg/kg) of heroin (blue line), 6-MAM (red line), or morphine (green line). **A** The total concentration of the injected opioid and its metabolites. Thus, the blue line represents the sum of heroin, 6-MAM, and morphine; the red line represents the sum of 6-MAM and morphine; the green line represents the sole morphine. **B** The concentrations of Dopamine in the same animals. **C** The ratio between the concentration of dopamine (DA) and the cumulative concentration of opioids: blue line = DA/(heroin+6-MAM + morphine), red line = DA/(6-MAM + morphine); green line DA/morphine. Based on data extrapolated from Gottås et al. 2014 [20] (same animals of Fig. 4).

In the rat, after i.v. heroin administration, morphine concentrations peak at 10–12.6 min in the blood and at 24 min in the striatum (supplementary material in [31]) and then decline very slowly (see Fig. 5).

Morphine metabolism mostly depends on its glucuronidation in the liver by two isoforms of the uridine 5’-diphospho-glucuronosyltransferase (UGT), UGT2B7 and UGT1A1 [57], although extra-hepatic metabolism has been described [58]. Morphine glucuronidation yields M6G and morphine-3-glucuronide (M3G).

Following heroin i.v. administration and inhalation in humans the ratio of M6G/M3G formation is about 1:6–8 [25]. In contrast, in the rat, morphine glucuronidation yields, under normal conditions, almost exclusively M3G [59]. However, repeated exposure to opioids can dramatically alter morphine glucuronidation both in humans and rats. Antonilli and colleagues [60] found higher concentrations of M6G and lower concentrations of M3G in people with heroin use disorder relative to heroin-naïve patients receiving morphine for pain control. It is unlikely that this phenomenon was due to the contaminants of street heroin, or to factors such as age, renal function, and route of administration, which are known to affect morphine metabolism [61, 62]. Increased synthesis of M6G after exposure to heroin can in fact be observed even in the rat (a species that physiologically synthetizes little or no M6G) using both in vivo (i.v. self-administration [63], non-contingent i.p. injections [64]) and in vitro procedures (liver microsomal preparation [63–65], primary cultures of hepatocytes [66]). This effect seems to be independent of mu opioid peptide receptors (MOP), as it was not reversed by the MOP antagonist naltrexone, nor mimicked by the MOP agonist methadone [65]. Antonilli and colleagues proposed that heroin might induce homo- or hetero-dimerization of UGTs at a post-translational level, depending on the substrate involved [66].

A minor metabolic pathway of morphine is represented by sulfation, yielding morphine-3-sulfate and morphine-6-sulfate. In humans, the plasma concentration of morphine-3-sulfate is several hundred times lower than that of M3G, while morphine-6-sulfate is undetectable in most people [67].

### Pharmacokinetics of M6G

Both M6G and M3G are excreted partly with the urine and partly with the stool, after biliary excretion [61]. A small fraction of M6G and M3G excreted with the bile is de-glucuronidated by enzymes produced by the colonic flora, and the resulting morphine undergoes enterohepatic cycling [53].

After heroin administration in humans, plasma concentrations of M6G peaks at around 1 h [21, 25] (Fig. 3), and then decline very slowly, with a t_1/2_ even longer than that of morphine (>3 h) [25]. The C_max_ in the plasma is similar to that of morphine [25, 47]. This could partially depend on a similarly low systemic clearance [22, 27] and on the scarce lipophilicity of M6G, which results in slow tissue distribution and low penetration of the blood brain barrier [68, 69]. A conformational molecular shift (folded vs un-folded), which increases M6G lipophilicity by masking the polar region, has been proposed to facilitate passive transport across the blood-brain barrier [70]. Furthermore, there is also evidence of active transport, not P-glycoprotein-mediated, at the blood-brain barrier [18]. Based on pharmacokinetic modelling, M6G blood-brain equilibration half-life was found to be approximately 7 h [71]. In the brain, extracellular levels of M6G are much higher than in the intracellular fluid [72]. Plasma protein binding of M6G is relatively low, being about 15% compared to 35% for morphine [68, 73]. Morphine glucuronides are detectable in urine up to 72 h post heroin administration.

## Pharmacodynamics of heroin and its metabolites

### Opioid receptors

The pharmacology of the endogenous opioid system is exceedingly complex and the readers interested in a thorough discussion of this topic are referred to the many authoritative reviews published in recent years (e.g., [74–76]) The goal of this section is simply to highlight some aspects of opioid receptor pharmacology that are relevant to the issues discussed in the following sections.

There are three main types of opioid receptors: µ (mu), *δ* (delta), and κ (kappa) opioid peptide receptors (MOP, DOP, KOP, respectively). Additional types have been proposed, like the nociceptin/orphanin FQ opioid peptide receptor, but their physiological/pharmacological status is still debated [76]. All opioid receptors are G_i/o_-protein-coupled receptors, whose canonical transduction cascade depends on the action of the α_i_ subunit (with inhibition of adenylyl cyclase and reduced synthesis of cAMP) and of the βγ subunits (resulting in reduced conductance of voltage-gated Ca^2+^ channels and the opening of rectifying K^+^ channels). This familiar view has been complicated over the years at several levels, particularly with respect to the MOP (alternatively named MOP, MOR, MOR1, OPRM, OPRM1). First, it was found that the MOP is coupled with alternative transduction mechanisms [e.g., β-arrestin-2 and protein kinase C, (PKC)], as a function of the ligand (‘biased agonism’) [77] and of the duration of the stimulation. Both β-arrestin-2 and PKC have been implicated in the development of tolerance (defined as the need to increase the dose to produce the same effect) after chronic exposure to MOP agonists [78–80]. Second, a variety of omo- and hetero-dimers (involving other types of opioid receptors as well as non-opioid G protein-coupled receptors) have been identified, each with distinctive transduction pathways. Third, the structure and expression of the MOP gene is very intricate, being characterized by extensive alternative splicing of coding exons. Pasternak and colleagues have long hypothesized that the complexity of splicing (which far exceeds that of receptor subtypes identified using pharmacological tools) might account for the qualitative and quantitative differences in the effects of MOP agonists and for the incomplete cross-tolerance among them [75, 81]. It is quite disconcerting that such an interesting hypothesis has not become the focus of more intense scrutiny. It is to be hoped that the recent opioid ‘epidemics’ in the USA serve as a stimulus to bring opioid pharmacology back to the forefront of addiction research, after several decades of almost exclusive focus on the pharmacology of psychostimulants and alcohol.

Table 2 provides a synopsis of pharmacodynamic parameters for heroin and its metabolites. Notice that these parameters were estimated using a variety of test assays, often in studies that did not compare all substances in parallel.

**Table 2:** Overview of the activity of heroin and its metabolites at opioid receptors.

			Heroin	6-MAM	Morphine	M6G	references
	MOP	affinity	<6MAM «M	>H < M	»H > 6MAM ≥ M6G	≤M	[12, 83, 84, 86, 109–112, 119, 120, 126]
		efficacy	=6MAM > M M6G	=H > M M6G	<H 6MAM ≤ M6G	<H 6MAM ≥ M	
		potency	«6MAM M M6G	»H = M M6G	»H = 6MAM ≤ M6G	»H = 6MAM ≥ M	
	DOP	affinity	<6MAM < M	>H > M	>H < 6MAM < M6G	≥M	[91, 92, 119, 120, 126–128]
		efficacy			=M6G	=M	
		potency			=M6G	=M	
	KOP	affinity	<6MAM M	>H = M	»M6G	«M	[109, 119, 127, 128]
		efficacy			=M6G	=M	
		potency			>M6G	<M	
Splice variants	MOP-1r	affinity			≥M6G	≤M	[112, 120, 121]
	MOP-2r	affinity			>M6G	<M	
	MOP gene exon1 KO		Analgesic activity retained	Analgesic activity retained	Analgesic activity not retained	Analgesic activity retained	[87] but see also [89, 90]
	MOP gene exon2 KO			Analgesic activity not retained	Analgesic activity retained	Analgesic activity not retained	[88] but see also [89]
	MOP gene exon11 KO		Analgesic activity not retained		Analgesic activity retained	Analgesic activity not retained	[166]

Affinity for the receptor was expressed either by the inhibitory constant Ki, that describes the binding affinity for a ligand that displaces a tracer saturation at the receptor, or by the half-maximal inhibitory concentration IC50, that is the concentration of a ligand that reduces the binding of a tracer to its receptor (or the biological activity of interest) by 50%. Efficacy was expressed by E_max_, that is the maximal response achievable from an agent/drug. Potency was expressed either by IC50 (see above) or EC50, intended as the concentration of a drug at which 50% of its maximum effect is achieved. To compare heroin and its metabolites, « and » indicate a value ten-fold less than or greater than; < and > a value less than 10-fold; ≤ and ≥ indicate that studies are not consistent and/or different test assays were used. Data from studies using antisense probes are not reported.

*H* heroin, *M* morphine, *6MAM* 6-monoacetylmorphine, *M6G* morphine-6-glucuronide, *MOP* mu opioid peptide receptor, *DOP* delta opioid peptide receptor, *KOP* kappa opioid peptide receptor, *KO* knock-out mice.

### Pharmacodynamics of heroin

In vitro studies have suggested that the affinity of heroin for the MOP is much lower than that of morphine and 6-MAM [82–84] (Table 2), which might depend on the lack of a free phenolic hydroxyl (3-OH) group in the molecular structure of heroin [85]. In contrast, it has been shown that heroin efficacy, as indicated by MOP-mediated G-protein activation, is higher than that of morphine and M6G, and at least comparable to that of 6-MAM [86]. In CXBK mice (a recombinant-inbred strain insensitive to morphine), as well as in mice treated with antisense probes targeting exon-1 of the MOP gene, the analgesic effect of morphine is suppressed while that of heroin and M6G is retained [87]; vice versa, antisense probes or knockout targeting exon-2 of the MOP gene minimize the analgesic effect of heroin without interfering with that of morphine [88]. However, these findings were not confirmed by other studies [89, 90].

### Pharmacodynamics of 6-MAM

As noted above, 6-MAM has greater affinity than heroin at MOP [82] but the same transduction efficacy, higher than that of downstream metabolites [86]. This might be due to their shared high affinity for the same splice variant of the MOP [88]. Furthermore, 6-MAM has affinity for the DOP, which might contribute to its potent analgesic effect [91, 92] (Table 2).

### Pharmacodynamics of morphine

Relative to 6-MAM or heroin, morphine has slightly higher affinity for the MOP [82] but lower efficacy in activating the G-protein cascade [86] (Table 2). Morphine binds, albeit with much lower affinity, also the DOP and the KOP. Indeed, some effects of morphine are thought to be mediated, at least in part, by DOP (supraspinal/spinal analgesia, respiratory depression, reduced gastrointestinal motility) and KOP (peripheral analgesia and dysphoria) [93, 94]. Furthermore, it has been proposed that the analgesic and respiratory depressant effects of morphine are mediated by distinct variants of the MOP (MOP-1r and MOP-2r, respectively) [95–97], although their existence is still controversial. The heroin:morphine analgesic potency ratio in humans has been estimated to be between 2:1 and 4:1 when administered by subcutaneous, i.v., or intramuscular injection [98–100] and 1.5:1 when given orally [101]. Comparative studies generally agree that heroin analgesia has faster onset but shorter duration than morphine analgesia [99, 102, 103]. The fact that heroin has a more favourable profile, in terms of adverse effects (e.g., nausea, respiratory depression, dysphoria) than morphine [103] (but see [99]) has led some authors to advocate the use of heroin in place of morphine in some clinical settings, although there is no consensus on this [99, 102, 104].

Chronic exposure to morphine results in tolerance to some of its effects (analgesia, euphoria, sedation, nausea, and respiratory depression), but not to others (e.g., constipation) [105, 106]. It is important to notice that tolerance to morphine can develop independent of the mechanisms responsible for the development of withdrawal syndrome. For example, PKC inhibition can reduce tolerance to the analgesic effects of morphine but does not prevent naloxone-precipitated withdrawal symptoms in mice [107]. Another important aspect of morphine tolerance is the limited cross-tolerance to heroin and 6-MAM, at least for what concerns analgesia [108].

### Pharmacodynamics of M6G

M6G selectively binds the MOP with a potency similar to that of morphine [109, 110], and its efficacy is thought to be equal to or slightly higher than that of morphine [86, 111]. Preclinical studies have shown much greater (13- to 808-fold, depending on the testing procedure and on the route of administration) analgesic response to M6G than to morphine [112–115]. In clinical studies, the analgesic efficacy of M6G appears to be at least equivalent, if not superior, to that of morphine [116]. Indeed, it has been shown that M6G is at least in part responsible for the analgesic effect of morphine [117, 118].

However, the affinity profile of M6G, relative to that of morphine, varies as a function of MOP subtypes [116, 119, 120]. As pointed out above, some studies suggest that in exon 1 MOP-1r gene knockout mice, the analgesic effect of M6G and heroin is retained, while morphine analgesia is suppressed [87]. In rats, preferential affinity for the MOP-1r relative to the MOP-2r subtype has been proposed as a possible explanation for the reduced degree of respiratory depression compared to morphine, but there are remarkable discrepancies in the findings of different studies [112, 120, 121] (Table 2). Other studies using antisense probes [122], as well as the selective antagonist 3-methoxylnaltrexone (3-MNTX) [123], have suggested the existence of a splice variant specific to M6G, as its analgesic action is antagonized without interference on MOP-, DOP- and KOP-mediated analgesia. Studies in clinical populations have shown that M6G has a more favourable profile than morphine with respect to nausea and vomiting [124, 125].

Morphine-6-glucuronide activity at DOP is approximately 6 times lower than at MOP, but similar if not greater than that of morphine [119, 126]. Activity at KOP seems to be negligible [127, 128].

In preclinical studies, no cross-tolerance to morphine was found with respect to analgesia [87] and locomotor activity [129], albeit the literature is not consistent [112]. Analgesic tolerance to M6G might depend on molecular adaptations similar to those of morphine (see above [130]). Finally, relative to morphine, M6G showed greater agonism bias for β-arrestin-2 over G-protein signalling [131].

### Pharmacodynamics of other metabolites

Morphine-3-glucuronide and morphine-3-sulfate seem to have no intrinsic pharmacological actions, but might behave like antagonists at MOP, as conjugation at the position 3’ may obstruct the binding of other ligands [26, 132]. The implications of this antagonism in modulating the response to heroin or morphine, especially after chronic exposure, is a controversial issue [133].

When administered intra-peritoneally in rats, morphine-6-sulfate exhibits much greater analgesic effect than morphine [134], suggesting that it may possess pharmacological activity similar to M6G. However, the contribution of morphine-6-sulfate to the effects of heroin must be negligible, as negligible are the plasma concentrations of this metabolite after administration of morphine or heroin in humans [67].

## Neurobehavioral effects of heroin and its metabolites

As mentioned in the previous sections, significant pharmacological activity of heroin metabolites has been demonstrated in several analgesia-related paradigms [29, 135, 136]. In contrast, other effects of heroin metabolites, possibly more relevant to heroin use disorder, have received much less attention.

### Blood oxygenation

The most serious side effect of MOP agonists is represented by respiratory depression and the ensuing brain hypoxia [137]. Kiyatkin and colleagues [138] thoroughly investigated the time course of the effects of heroin, 6-MAM, and morphine on oxygen levels in the rat nucleus accumbens (NAc), a brain area thought to play a crucial role in natural and drug reward [139, 140]. They found that heroin induces a biphasic change, with an initial decrease, produced by respiratory depression, followed by an increase, produced by vasodilation. The effects of 6-MAM followed a similar pattern, but the decrease in oxygen levels was more rapid and yet smaller than those observed after heroin. The most obvious explanation for the latter phenomenon is that the high permeability of the blood-brain barrier to heroin, coupled with rapid deacetylation by brain esterases, leads to 6-MAM concentrations greater than those that would result from the administration of equimolar doses of 6-MAM [20]. In contrast, morphine has little effect on NAc oxygen levels. Thus, it appears that 6-MAM plays a major role in producing the brain hypoxia associated with heroin overdose [137].

### Dopaminergic transmission

Dopaminergic transmission has long been thought to play a major role in drug reward [139, 140]. It is commonly assumed that all substances of abuse increase dopaminergic transmission [141, 142], albeit via different mechanisms of action. In particular, heroin and other MOP agonists are thought to increase dopamine concentrations in the terminal regions of the meso-striatal dopaminergic system by binding MOP located on inhibitory GABAergic neurons, hence disinhibiting dopamine neurons [143–145]. Yet, experimental evidence does not support the notion that the rewarding effects of heroin are mediated by dopaminergic transmission.

In the first place, there is no evidence that heroin increases dopamine transmission in humans. Daglish and colleagues [146] and Watson and colleagues [147] found in fact, using [^11^C]raclopride positron emission tomography (PET) imaging in people with heroin use disorder, that heroin’s high is not associated with changes in dopamine receptor binding in the striatum. Another PET imaging study conducted in a small sample of non-opioid-dependent people found that morphine produces a very small increase in dopamine receptor occupancy (about 8%), which, however, is inversely correlated with subjective ratings of ‘high’ and other measures of reward [148].

Likewise, research in rodents has failed, so far, to provide direct evidence of a causal link between heroin reward and dopamine levels. Microdialysis experiments in the rat have shown that i.v. administration of heroin can increase extracellular dopamine concentrations over a time scale of several minutes (e.g., [149, 150]). However, only modest [151] or negligible [149] changes in dopamine concentrations were observed during self-administration (the gold standard for the investigation of the reinforcing effects of addictive drugs; see below). Even more perplexing are the findings from voltammetric studies, which allow to monitor dopaminergic activity on a second or sub-second scale. A sharp decrease in the dopamine signal was observed immediately after self-administered or experimenter-administered i.v. injections of heroin [152, 153]. Similarly, electrophysiological experiments by Kiyatkin and Rebec (1997) [154] have shown a transient inhibition of dopaminergic neurons in association with heroin self-administration. Finally, experiments conducted over the last three decades have repeatedly shown that disruption of dopaminergic transmission (via lesions or receptor blockade/silencing) has little or no effect on heroin or morphine self-administration in the rat [155–163]. Against this profusion of ‘negative’ findings in the rat, stand the results of studies conducted using optogenetic tools in mice, which implicate dopaminergic mechanisms in heroin self-administration [164, 165]. It is worth noticing that the interpretation of these findings is complicated by the difficulty of extricating the pharmacological effects of drugs from the response to conditioned stimuli paired with drug administration or self-administration.

To the best of our knowledge, the effect of heroin metabolites on dopaminergic transmission has been investigated only by Gottås and colleagues [31]. These authors quantified plasma and striatal concentrations of heroin, 6-MAM, morphine, and dopamine in rats that had received i.v. injections of equimolar doses of heroin, 6-MAM, or morphine (see Figs. 4 and 5). After 6-MAM, striatal dopamine increased in apparent temporal relationship with striatal 6-MAM (both with T_max_ = 8 min). However, dopamine levels increased with a delay of 3–4 min relative to those of 6-MAM and then declined sharply (striatal t_1/2_ ≈ 12 min), far in advance of the much slower decline of 6-MAM (striatal t_1/2_ ≈ 24 min). Completely different was the pattern produced by morphine. After an injection of morphine, striatal concentrations of dopamine increased much more slowly (T_max_ = 46 min) than those of morphine (T_max_ = 6 min). Heroin produced a third pattern: while striatal heroin peaked rapidly (T_max_ = 2 min), dopamine began to rise only at 8 min (panel B, Fig. 5) and peaked at 14 min, after heroin had all but disappeared. Furthermore, the C_max_ of striatal dopamine was much smaller after heroin (approximately 5 nM) than after 6-MAM (approximately 10 nM), even though, as discussed in a previous section, the striatal concentrations of 6-MAM were about 50% higher after an injection of heroin than after an equimolar dose of 6-MAM. It is remarkable that the ratio of dopamine to total opioid concentrations (that is, the sum of the concentration of the parent drug and its metabolites) in the striatum was much smaller after heroin than after 6-MAM and followed a completely different temporal profile (panel C, Fig. 5).

One possible interpretation for these findings rests on the dynamics of agonist-receptor interactions. The contrasting effects of 6-MAM vs. heroin on dopamine release might depend on distinct patterns of action at MOP, DOP, and KOP, similar to what was seen for the antinociceptive activity in different strains of mice [92]. Heroin, for example, might act on a splice variant of the MOP [81, 166], possibly with regulatory actions on other opioids and/or receptor types.

In any case, the fact that heroin, 6-MAM, and morphine produce distinct and contrasting effects on dopamine release challenges simple assumptions concerning the relationship between the rewarding effects of MOP agonists and dopamine transmission (see Conclusions).

### Psychomotor activity

Ever since the seminal paper by Wise and Bozarth [139], many studies have investigated drug-induced psychomotor activity as a proxy for drug reward, and drug-induced psychomotor sensitization as process homologous to the development of drug addiction, at least in animal models [140]. Despite considerable evidence that does not support this notion (e.g., [167–170]), there is still a great deal of interest in the psychomotor effects of addictive drugs in rodent models [162].

With respect to heroin [171], 6-MAM is nearly equipotent in inducing naloxone-reversible psychomotor activity [172–174] and psychomotor sensitization following repeated administration [175]. Although morphine is considerably less potent than heroin and 6-MAM in enhancing locomotor activity [174], it can easily induce psychomotor sensitization [176, 177].

Increased but delayed locomotor activity has been reported also for M6G [178], although the total distance travelled following M6G was lower than after equimolar doses of morphine, and repeated administrations produced MOP-dependent psychomotor sensitization [179, 180]. Remarkably, the locomotor response to a combination of morphine and M6G was not greater than that observed after morphine alone [178], and while pre-treatment with morphine sensitized the locomotor response to M6G, pre-treatment with M6G did not sensitize the response to morphine [180]. These results indicate that all metabolites might contribute to the locomotor-sensitizing effects of heroin, maybe with distinct mechanisms of action, as suggested by the incomplete cross-sensitization between M6G and morphine.

### Drug discrimination

Few studies have directly compared the subjective effects of heroin and morphine in humans. Martin and Fraser [181] administered, using a double-blind design, equianalgesic doses of the two drugs to opiate-experienced users. After an acute injection, morphine and heroin did not differ in self-reported effects, such as relaxation, itchy skin, nausea, and sleepiness. Remarkably, when asked to guess which of the two drugs the participants had received, morphine was recognized with more accuracy than heroin.

In preclinical studies, heroin was successfully used as a training drug in discriminative procedures. It was shown that in the rat the discriminative stimulus properties of 6-MAM and morphine overlap with those of heroin [182]. However, while the response rate for 6-MAM was comparable to that observed after equimolar doses of heroin, 10-fold higher doses were required for morphine. Similar findings were obtained in monkeys [183].

In a drug discrimination procedure, M6G was fully substituted for heroin in rhesus monkeys [184] and for morphine in rats [185]. In the latter study, M6G was 17 times more potent than morphine when these drugs were administered in the cerebral ventricles, but less potent when injected subcutaneously. The discriminative effects of the two heroin metabolites were blocked by naltrexone. The putative M6G receptor antagonist 3-MNTX did not differentially modulate the discriminative stimulus of heroin, morphine and M6G [186]. Overall, it is tempting to assume that all heroin metabolites are equipotent in mediating the interoceptive effects of the parent compound by acting on the same receptors.

### Electrical brain self-stimulation

The propensity of rats to electrically self-stimulate discrete brain regions (particularly the lateral hypothalamus — that is, the medial forebrain bundle) was discovered in the mid-1950s by Olds and Milner [187]. Since then, intracranial self-stimulation (ICSS) has been widely used to investigate the effects of drugs of abuse on the putative neural substrates of reward. Heroin was shown to facilitate ICSS of the lateral hypothalamus when administered i.p. at high doses [188] and i.v. at doses that maintain self-administration [189]. The same effect had been previously described for morphine [190, 191], with no tolerance developing to it even after several weeks of intermittent treatment at increasing dosage [192].

In the early 1990s, Hubner and Kornetsky compared the effects of heroin, 6-MAM, and morphine on the threshold for ICSS of the medial forebrain bundle [193], and found that 6-MAM was as potent as heroin and about 40 times more potent than morphine. The same study also demonstrated that heroin and 6-MAM were equipotent (and 6.5 times more potent than morphine) in raising the escape threshold for the aversive stimulation of the mesencephalic reticular formation. No study has yet tested M6G in the ICSS paradigm.

### Conditioned place preference (CPP)

When animals are repeatedly exposed to one of the two chambers of a CPP apparatus while under the effects of an addictive drug, they exhibit a preference for the drug-paired chamber relative to the vehicle-paired chamber [194–196]. The preference for the drug-paired context is commonly considered a measure of the rewarding effects of the drug, albeit the limitations of this procedure have been noted by many authors [169, 194, 195]. It has been known for a long time that morphine and heroin can induce CPP in monkeys and rodents [197–206], heroin being 10 times more potent than morphine in this respect [207]. More recently, the same phenomenon was observed with 6-MAM [175] and M6G [208–210]. There is some evidence that heroin- and morphine-induced CPP might be mediated by these two metabolites. Pre-treatment with monoclonal antibodies (mAb) raised against 6-MAM has been shown to block the development of both 6-MAM- and heroin-induced CPP (although a higher titer was required to block heroin-induced CPP) [211]. When administered intracerebroventricularly, M6G was about 150 times more potent than morphine [210]. This effect was naloxone reversible.

### Self-administration

A great deal of research has investigated the reinforcing effects of heroin and morphine using i.v. self-administration procedures in primates and rodents. These studies have shown greater reinforcing potency of heroin relative to morphine [212, 213]. This is consistent with the slower onset of action of morphine relative to heroin [72, 73, 214] and matches the anecdotal preference for heroin over morphine reported by opioid users [215]. More pronounced adverse effects for morphine vs. heroin [216] or for heroin vs. morphine [217] were reported. However, in the already mentioned study by Martin and Fraser [181], when equianalgesic doses of heroin and morphine were administered double-blind to opiate-experienced users, no difference in desirable effects (including euphoria, relaxation, and ‘drive’) was reported. Current and/or past drug history of experimental subjects, the precise definition of subjective states such as ‘euphoria’ or ‘pleasure’, and the circumstances surrounding drug use [218] are some of the variables to consider when comparing human and animal data.

Self-administration of 6-MAM in the rat has been investigated only recently by Avvisati and colleagues [219]. This study compared equimolar doses of 6-MAM and heroin, showing that 6-MAM can sustain self-administration at even higher rates than heroin (even though the lack of complete dose-effect curves and of progressive ratio procedures makes it difficult to compare the relative rewarding efficacy of the two drugs). Furthermore, using an established rat model of relapse [220], the same authors were able to show that, like heroin, 6-MAM could trigger drug-seeking after a period of abstinence. These data suggest that 6-MAM has intrinsic addictive potential and might mediate at least some aspects of heroin reward. However, anti-6-MAM mAb, although effective in blocking the reinstatement of 6-MAM seeking, failed to prevent relapse into heroin seeking and re-acquisition of responding for the drug [219]. This cannot be attributed to a generalized inability to block the effects of 6-MAM, as anti-6-MAM mAb have been shown to blunt heroin-induced psychomotor activity [173, 211] and CPP [175], which is not surprising, given that many studies have shown dissociation among these effects of heroin [167–170]. It is still possible that the quote of 6-MAM formed in the brain (which cannot be affected by peripheral anti-6-MAM mAb) might be sufficient to sustain self-administration, explaining the lack of effect of the anti-6-MAM mAb. Indeed, brain levels of 6-MAM are reduced by anti-6-MAM mAb to a lesser extent after heroin administration than after 6-MAM administration [173].

Pharmacokinetic modelling suggests that brain concentrations of 6-MAM might contribute to determine the pattern of heroin self-administration in the rat. In a recent study in rats, two schedules of heroin self-administration were compared. One group of rats were trained with a 20-s timeout after each heroin infusion, whereas the other rats were trained without timeout. The rats in the latter group self-administered heroin in a ‘burst-like’ pattern, which produced spikes in 6-MAM that were sharper and of greater magnitude than those observed in rats trained on a timeout schedule. Interestingly, the drops in 6-MAM concentrations coincided with the resumption of lever pressing for heroin [221].

To date, no study has directly investigated the rewarding effects of M6G in either humans or animals. And yet, as pointed out in previous sections, there is evidence of increased M6G synthesis in people with heroin use disorder [60]. It has been shown that heroin self-administration can induce the synthesis of M6G in the rat, as indicated by increased levels of M6G in rats that had self-administered heroin relative to those that had self-administered saline [63]. Likewise, the V_max_ for M6G synthesis in liver microsomal preparations incubated with morphine, correlated with plasma levels of M6G in rats that had self-administered heroin, whereas M6G was undetectable in rats that had self-administered saline. Furthermore, in rats trained to self-administer heroin or morphine, the M6G receptor antagonist 3-MNTX increased the infusion rate of both drugs at doses effective in blocking morphine analgesia only [123, 222]. These findings point to a possible role of M6G in the reinforcing effects of heroin, although the exact pharmacological mechanism (e.g., a possible M6G-specific receptor subtype) remains elusive. Finally, studies in rodents have shown that increased synthesis of M6G might potentiate heroin withdrawal syndrome. Naloxone-precipitated symptoms are in fact more severe after repeated M6G administration than after morphine [128].

## Distinct roles for heroin and its metabolites in heroin addiction

We reviewed here major aspects of the neuropsychopharmacology of heroin and of its active metabolites: 6-MAM, morphine, and M6G. While it is often assumed that the rewarding effects of heroin depend on morphine, the contribution of 6-MAM and M6G have not yet received enough attention. Indeed, there is growing evidence that heroin and its three metabolites all play a major role in both the acute effects of heroin, and in the development of heroin use disorder.

### Heroin, 6-MAM and the heroin ‘flash’

After i.v. injection in humans, heroin peaks at 30 s in the arterial blood (and presumably in the brain) [26], a timing synchronous with the characteristic heroin ‘flash’, highly desired by most users [14, 15]. Although 6-MAM is produced by plasma esterases while heroin is still distributing to the brain and other peripheral compartments, heroin remains by far the prevailing opioid in the plasma for about 8 min [26]. An in-vitro study using crude membrane preparations from rat brains has shown that the affinity of heroin to MOP is lower than that of 6-MAM [23], suggesting that even in the presence of higher concentrations of heroin, MOP would preferentially bind 6-MAM. However, it is not clear to what extent this finding would translate to humans, both because of the specific binding site labeled in this study (which did “not allow recognition of the various opioid receptor subtypes”), and because of possible species-specific differences. Thus, to the extent that during the first minutes after i.v. injection, heroin and 6-MAM coexist in the blood and in the brain, there is no reason to dismiss the role of heroin itself in producing the ‘flash’.

One way to untangle the contribution of heroin versus 6-MAM would be to block the deacetylation of heroin. Peripheral administration of the cholinesterase inhibitor tri-ortho-tolyl phosphate (which does not cross the blood-brain barrier) was found to increase the analgesic potency of heroin (but not that of 6-MAM or morphine) in the mouse [83]. Yet, the same study found that in vitro inhibition of tissue esterases in membrane preparations from the rat brain reduced MOP occupation, suggesting that the increased analgesic effect of heroin might depend on its deacetylation to 6-MAM in the brain. It remains to be determined whether similar manipulations of enzymatic activity would also affect the rewarding effects of heroin both in animals and in humans.

Another appraoch would be to compare the brain concentration profiles of heroin and 6-MAM with the time-course of early neurobiological effects. Of course, this type of studies cannot be conducted in humans, at least at this time, but only in experimental animals. As discussed in detail in previous sections, Gottås and colleagues [31] found that i.v. administration of 6-MAM in the rat increases dopamine concentrations in the rat striatum, with a temporal profile somewhat related to the temporal profile of the striatal concentrations of 6-MAM itself. However, and quite surprisingly, the C_max_ of striatal dopamine after heroin was half that observed after 6-MAM, with a much longer T_max_ (14 min after heroin vs. 8 min after 6-MAM). This suggests that the pharmacological actions of heroin somewhat ‘antagonize’ those of 6-MAM, at least for what concern dopamine release in the rat striatum. In any case, to the extent that the kinetics of dopamine release observed in the rat [31] reflect those occurring in humans, it appears that the temporal pattern of striatal dopamine does not match the almost instantaneous ‘flash’ reported by heroin users after i.v. administration [25, 47, 147], consistent with the literature that questions the contribution of dopamine transmission to heroin reward [222–225]. Dopamine-independent mechanisms of heroin reward have been proposed [226, 227], although this area of research is still inexplicably understudied.

### Contribution of morphine and M6G to heroin reward

Both morphine and M6G have been shown to possess intrinsic rewarding effects. Morphine produces subjective effects similar to those of heroin when given i.v. [181, 228], and while no such comparative studies have yet been performed for M6G, it has been reported that this metabolite is at least as effective as morphine in inducing CPP [210]. Thus, while 6-MAM is undoubtedly the main metabolite present in the first few minutes following heroin administration, it is reasonable to assume that also morphine and M6G play a part in the subjective and behavioral effects of heroin. Morphine concentrations surpass those of heroin and 6-MAM at about 10 min after i.v. administration of heroin [22, 25] (Fig. 3), and M6G concentrations surpass those of 6-MAM at about 25 min [25]. Although the temporal profiles of morphine and M6G concentrations do not match the heroin ‘flash’, they overlap with the subsequent, long-lasting feelings of contentment, or ‘tranquil-high’, which is much appreciated at least by some users [13, 229].

### Morphine- and M6G-induced neuroadaptations

The long half-lives of morphine (about 1.5–3 h) and M6G (about 2–6 h) might play a role not only in the acute rewarding effects of heroin but also in the neural adaptations underlying tolerance, physical dependence, and transition to addiction [230, 231]. Morphine and M6G slow clearance from the brain coupled with their preferential extracellular distribution produce in fact prolonged activation of MOP [116]. The pharmacological actions of M6G [90] might be of particular relevance, as indicated by their high efficacy at MOP located in the locus coeruleus [111], where opioid-induced long-term neuronal inhibition is an important contributor to the development of tolerance [232, 233]. The high efficacy of M6G in activating the β-arrestin-2 pathway has been implicated in the vulnerability to side-effects such as respiratory depression [234], though this hypothesis has been challenged by recent studies [235–237]. Like morphine and other opioid agonists (including those used as replacement treatments: buprenorphine and methadone [238, 239]), M6G can produce hyperalgesia and this effect is more robust and of more rapid onset than with morphine [240]. Hyperalgesia is a characteristic feature of heroin withdrawal syndrome [241] and seems to predict the intensity of heroin craving induced by drug cues [242]. Indeed, two preclinical studies have suggested a putative role for M6G in potentiating opioid withdrawal symptoms [128, 243].

In the light of the findings summarized above, it is interesting that higher M6G/M3G concentration ratio was found in people with heroin use disorder relative to people receiving morphine for pain control [60]. Furthermore, late clinical impairment in heroin users was found to be associated with higher levels of M6G [244], and M6G accumulation was observed in morphine-treated patients with renal failure [245]. Thus, it would be important to investigate the possible role of M6G in producing the neuroplastic adaptations implicated in the development of heroin addiction, as well as associated pathologies.

### Four tentative questions

Human studies are sorely needed to tease apart the role of heroin and its metabolites (as well as the interaction among them) in heroin addiction. At present, we know very little about the reinforcing effects of 6-MAM and M6G, and what we know comes exclusively from animal studies (see sections on ICSS, CPP, and self-administration). Thus, it would be important to investigate the rewarding effects of these two metabolites in humans. Furthermore, future research should address four crucial questions. Is the initial, instantaneous, short-lived ‘flash’ produced by i.v. heroin due to the similarly instantaneous spike in arterial concentrations of heroin (implying a similarly instantaneous spike in the brain)? Is the subsequent pleasant state of stunned calm the result of 6-MAM actions? Is the prolonged sense of contentment and well-being experienced by some heroin users the result of morphine and/or M6G actions? Do morphine and M6G contribute to the neuroplastic adaptations responsible for heroin use disorder and vulnerability to relapse? These issues might be addressed by testing the effects of enzymatic inhibitors, as well as of compounds that mimic the pharmacodynamic profiles of heroin, 6-MAM, or morphine without being subject to enzymatic transformation in active metabolites.

## Conclusions

For a long time, morphine has been considered the substance responsible for the rewarding effects of heroin, the latter being demoted to the status of pro-drug. In this review we argued for a more complex interplay among four actors: heroin per se, 6-MAM, morphine, and M6G. This novel viewpoint has important clinical and theoretical implications.

Given its chronic relapsing nature, the treatment of heroin use disorder is mainly based on long-term replacement treatment with opioid agonists such as oral methadone and sublingual buprenorphine [246]. Both drugs possess clinically favourable pharmacokinetic and pharmacodynamic profiles: higher affinity to MOP than morphine [247, 248], high brain uptake [249, 250], long duration of action [11]. Buprenorphine also act as a neutral antagonist at DOP and KOP, and being a partial agonist with low dissociation constant at MOP, behaves as an antagonist towards heroin [251, 252]. Methadone and buprenorphine replacement treatments are extremely effective in most clients [253, 254]. However, these treatments present some limitations. The retention rate is not optimal, with a drop-out rate of 40–60% within the first 12 months (often because of side effects such as dysphoria or QT interval prolongation [255]). Hence the search for alternative therapeutic options. Several European countries and Canada have approved the use of slow-release oral morphine, which seems to be more effective than methadone in reducing craving [256–258]. Similarly effective are the immediate or extended release oral formulations of diacetylmorphine [48, 259], as well as diacetylmorphine nasal spray [42]. The same rationale obviously applies to heroin-assisted treatment (HAT) programs, which appeal to a subgroup of users otherwise resistant to usual replacement treatments [260, 261]. The fact that pharmaceutical grade heroin and morphine already represent viable replacement strategies for heroin use disorder, suggests that also 6-MAM and M6G, or compounds that mimic their distinctive pharmacokinetic/pharmacodynamic profiles, should become the focus of research aimed at developing diversified and individualized treatment programs. Clinicians have long recognized the existence of major individual differences in the response to the many MOP agonists available for pain control [262]. It is in fact impossible to predict for each client the optimal drug and/or the optimal dosage, in terms of both analgesic efficacy and adverse effects. What works well in one person might not do so in another. There is no reason to think that this line of reasoning should not apply to opioid replacement treatment.

Finally, we have emphasized the role of dopamine-independent mechanisms in heroin reward, without discounting the possible contribution of dopamine-dependent mechanisms, particularly in the case of 6-MAM. In this respect it is important to notice that major differences in the ability to engage dopaminergic transmission are not limited to heroin and its metabolites. Even more dramatic differences are evident when opiates like morphine are compared to synthetic opioids, such as oxycodone [263]. Therefore, the pharmacological mechanisms responsible for the rewarding effects might differ greatly from one opioid agonist to another, particularly in terms of the involvement of the dopaminergic system. Lumping all opioid agonists under a single label might hinder a better understanding of opioid use disorders. Heroin and its metabolites represent a unique combination of opioids with partly different mechanisms of action. It follows that heroin users do not take a single drug but four (heroin, 6-MAM, morphine, and M6G), each with its distinctive rewarding profile, which might help to explain why heroin is still the most abused opioid world-wide.
